# Identification of novel pathway partners of p68 and p72 RNA helicases through Oncomine meta-analysis

**DOI:** 10.1186/1471-2164-8-419

**Published:** 2007-11-15

**Authors:** Brian J Wilson, Vincent Giguère

**Affiliations:** 1 Molecular Oncology Group, Room H5-45, McGill University Health Centre, 687 Pine Avenue West, Montréal, Québec, Canada

## Abstract

**Background:**

The Oncomine™ database is an online collection of microarrays from various sources, usually cancer-related, and contains many "multi-arrays" (collections of analyzed microarrays, in a single study). As there are often many hundreds of tumour samples/microarrays within a single multi-array results from coexpressed genes can be analyzed, and are fully searchable. This gives a potentially significant list of coexpressed genes, which is important to define pathways in which the gene of interest is involved. However, to increase the likelihood of revealing truly significant coexpressed genes we have analyzed their frequency of occurrence over multiple studies (meta-analysis), greatly increasing the significance of results compared to those of a single study.

**Results:**

We have used the DEAD-box proteins p68(Ddx5) and p72(Ddx17) as models for this coexpression frequency analysis as there are defined functions for these proteins in splicing and transcription (known functions which we could use as a basis for quality control). Furthermore, as these proteins are highly similar, interact together, and may be to some degree functionally redundant, we then analyzed the overlap between coexpressed genes of p68 and p72. This final analysis gave us a highly significant list of coexpressed genes, clustering mainly in splicing and transcription (recapitulating their published roles), but also revealing new pathways such as cytoskeleton remodelling and protein folding. We have further tested a predicted pathway partner, RNA helicase A(Dhx9) in a reciprocal meta-analysis that identified p68 and p72 as being coexpressed, and further show a direct interaction of Dhx9 with p68 and p72, attesting to the predictive nature of this technique.

**Conclusion:**

In summary we have extended the capabilities of Oncomine™ by analyzing the frequency of coexpressed genes over multiple studies, and furthermore assessing the overlap with a known pathway partner (in this case p68 with p72). We have shown our predictions corroborate previously published studies on p68 and p72, and that novel predictions can be easily tested. These techniques are widely applicable and should increase the quality of data from future meta-analysis studies.

## Background

Recently there have been attempts to correlate published microarrays, using software that can analyze many thousands of microarrays at one time. One such program is called Oncomine™ [1], where each study within Oncomine™ is in essence a collection of individual microarrays from many patient samples[2]. These "multi-arrays" usually utilise either normal or tumour biopsy samples (or compare both together), from various tissue sources.

**Figure 1:**
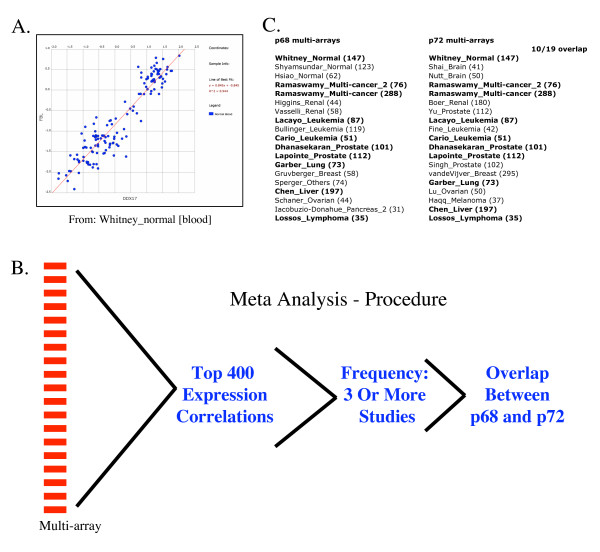
**Oncomine studies utilised and methodology of analysis**. (A) Screenshot example of Oncomine™ output of p72 (*DDX17*) coexpression with fibrillarin (*FBL*) in one multi-array study, covering 147 samples. p72 is X-axis and fibrillarin is Y-axis. (B) Procedure employed for meta-analysis of 19 different multi-arrays after searching for either p68 or p72, extracting the top 400 coexpressed genes from each multi-array, and comparing for frequency of repetition. (C) Chosen multi-arrays to be studied for both p68 and p72.

One function of Oncomine™ is a search tool where the user's chosen gene is correlated in expression, within multi-arrays, with other genes in the array (both high and low expression, over all the samples in the multi-array). For example searching p72 (*DDX17*) gives several correlations in many multi-arrays. Focusing within the study Whitney_normal there is a high correlation with expression of fibrillarin, over the 147 blood samples tested (Figure 1A). In samples where p72 expression was diminished, so was fibrillarin, and conversely when p72 expression was high, so is that of fibrillarin. This result is made more significant given that p72 and fibrillarin have previously been shown to interact together[3].

Correlations like this can show if proteins may be in the same pathway (e.g. both coregulated together, or one directly affecting the other), although it cannot show more than association. In an attempt to further increase the stringency of Oncomine™ to elude to these pathways we chose to test the DEAD-box proteins p68 and p72 because they are highly similar proteins that interact together and have been shown to be involved in defined cellular functions including splicing and transcription, which can then be used as a quality control measure of this technique [4-10]. Also as p68 and p72 are so similar there is the possibility that they may to some extent be functionally redundant.

In total this means that we can perform a meta-analysis of p68 coexpressed genes independent to that of p72, then compare the results for overlap (Figure 1B). If the gene lists were to give a significant overlap then this would act to support the notion that the technique is highly selective. Our results reveal that, not only does this technique corroborate previously published data on p68 and p72, it also generates testable predictions of novel pathway partners of p68 and p72.

## Results

### Overlapping coexpressed genes of p68 and p72

Multi-arrays chosen for meta-analysis had many individual samples/microarrays, indicating that a good correlation coefficient given by Oncomine™ is already highly significant. Figure 1C indicates the chosen multi-array studies for p68 and p72. Note that there is almost a 50% overlap of studies chosen.

Meta-analysis results, with frequency of 3 or more, for p68 yielded a higher volume of hits than for p72 (see Additional file 1). Both of these lists were compared for common genes and the common list was further assessed for ontology and full gene names (Table 1). Remarkably, we observed a large number of overlapping genes, indicative of the stringency employed in this technique.

**Table 1:** Frequency overlap between p68 and p72 coexpressed genes.

Gene	p68 %	p72 %	Function	Gene Name
TIA1	26%	16%	Splicing	cytotoxic granule-associated RNA-binding protein
SFRS5	**37%**	21%	Splicing	splicing factor, arginine/serine-rich, 5
SFPQ	**42%**	**47%**	Splicing	splicing factor proline/glutamine rich (polypyrimidine tract binding protein associated)
SF1	**37%**	26%	Splicing	splicing factor 1
MBNL1	**53%**	**32%**	Alternative Splicing	muscleblind-like protein(Triplet-expansion RNA-binding protein)
HNRPH1	**47%**	**32%**	Splicing	heterogeneous nuclear ribonucleoprotein H (hnRNP H)
CROP	21%	**37%**	SR Protein -Splicing?	cisplatin resistance-associated overexpressed protein (LUC7A)
CPSF2	**42%**	21%	Splicing	cleavage and polyadenylation specificity factor
C6orf111	**32%**	**32%**	Splicing	splicing factor, arginine/serine-rich 130
FLJ12529	21%	16%	Splicing	pre-mRNA cleavage factor I, 59 kDa subunit
DDX5	100%	26%	Splicing/Transcription	p68 DEAD-box RNA helicase
DDX17	26%	100%	Splicing/Transcription	p72 DEAD-box RNA helicase
PAPOLA	26%	21%	Transcription/Splicing	poly(A) polymerase alpha
ILF3	26%	21%	Transcription/Splicing	NFAR1/NF-90/subunit of NFAT transcription factor
PNN	16%	21%	Transcription/Splicing	pinin(DRS)
XBP1	26%	21%	Transcription/ER-alpha pathway	X-box binding protein 1
THRAP2	**32%**	21%	Transcription?	thyroid hormone receptor associated protein 2
RORA	26%	26%	Transcription	RAR-related orphan receptor alpha
PTMA	21%	16%	Transcription	prothymosin, alpha (gene sequence 28)
DHX9	**47%**	**32%**	Transcription	RNA Helicase A/DEAH (Asp-Glu-Ala-His) box polypeptide 9
BMI1	21%	16%	Transcription Silencing	B lymphoma Mo-MLV insertion region (mouse) [Polycomb complex protein BMI-1]
SMARCA2	16%	21%	Transcription	SWI/SNF related, matrix associated, actin dependent regulator of chromatin, subfamily a, member 2
HIF1A	16%	16%	Transcription	hypoxia-inducible factor 1, alpha subunit (basic helix-loop-helix transcription factor)
MAP3K7IP2	26%	21%	Signal Transduction/Transcription	mitogen-activated protein kinase kinase kinase 7 interacting protein 2 (TAB2)
PRKAR1A	**47%**	**32%**	Signal Transduction	protein kinase, cAMP-dependent, regulatory, type I, alpha (tissue specific extinguisher 1)
PIK3R1	21%	21%	Signal Transduction	phosphoinositide-3-kinase, regulatory subunit 1 (p85 alpha)
HIPK2	**32%**	16%	Signal Transduction/Apoptosis	homeodomain interacting protein kinase 2
DNAJC3	21%	16%	Signal Transduction	DnaJ homolog subfamily C member 3 (Interferon-induced, double-stranded RNA-activated protein kinase inhibitor)
CSNK1A1	**32%**	21%	Signal Transduction	casein kinase 1, alpha 1
GNAS	21%	21%	Receptor-Stimulated G-Protein	guanine nucleotide binding protein (G protein), alpha stimulating activity polypeptide 1
ABI2	26%	21%	Cytoskeleton	Abl-interactor 2 (Abelson interactor 2)
ARPC3	21%	26%	Cytoskeleton	actin related protein 2/3 complex, subunit 3, 21kDa (p21-ARC)
FNBP4	26%	21%	Cytoskeleton?	formin binding protein 4
WASPIP	16%	16%	Cytoskeleton	Wiskott-Aldrich syndrome protein interacting protein
UTRN	16%	21%	Cytoskeleton	utrophin (homologous to dystrophin)
RAP2A	16%	16%	Cytoskeleton?	RAP2A, member of RAS oncogene family
NEDD5	16%	21%	Cytoskeleton/cell-cycle?	septin 2 (GTP-binding protein family)
ACTB	16%	26%	Cytoskeleton	beta actin
MAPRE2	16%	16%	Cytoskeleton	microtubule-associated protein, RP/EB family, member 2
SDCBP	21%	21%	Scaffold Protein	syndecan binding protein (syntenin)
HNRPU	**42%**	**32%**	Nuclear Matrix Attachment	heterogeneous nuclear ribonucleoprotein U (scaffold attachment factor A)
XPO1	26%	21%	Nuclear Export	exportin 1 (CRM1 homolog, yeast)
TNPO1	26%	32%	Nuclear Import	transportin 1
NUP133	26%	16%	Nuclear Pore	nuclear pore complex protein Nup133
ZFR	26%	21%	Nuclear RNA binding	zinc finger RNA binding protein
RAB5A	16%	16%	Endocytosis	RAB5A, member RAS oncogene family
RAB6A	**68%**	16%	Golgi-ER trafficking	RAB6A, member RAS oncogene family
GDI2	26%	26%	ER-golgi?(Interacts Rab6, above)	rab GDP-dissociation inhibitor, beta
EDEM1	21%	**32%**	Calnexin cycle/protein folding	ER degradation enhancer, mannosidase alpha-like 1
RAB14	**32%**	26%	Golgi-endosome trafficking	RAB14, member RAS oncogene family
PLEKHB2	26%	16%	post-golgi vesicle protein	pleckstrin homology domain containing, family B (evectins) member 2
TMP21	**37%**	**37%**	Trafficking	transmembrane trafficking protein
TRAM1	26%	16%	Protein Translocation	translocation associated membrane protein 1
SLC38A2	21%	21%	Amino acid transport	Solute carrier family 38, member 2
SLC25A5	26%	25%	ADP/ATP carrier protein	Solute carrier family 25 (mitochondrial carrier; adenine nucleotide translocator), member 5
CGI-109	**37%**	16%	Protein transport?	hypothetical protein
USP9X	21%	16%	Ubiquitin	ubiquitin specific protease 9, X chromosome (Drosophila fat facets related)
UBE2J1	21%	**32%**	Ubiquitin	ubiquitin-conjugating enzyme E2, J1 (UBC6 homolog, yeast)
UBE3A	16%	21%	Ubiquitin	ubiquitin protein ligase E3A
BIRC6	16%	21%	Ubiquitin ligase/Anti-apoptosis	baculoviral IAP repeat-containing 6 (apollon)
BIRC2	**32%**	21%	Apoptosis-resistance	baculoviral IAP repeat-containing 2
PSMA2	21%	21%	Proteasome	proteasome (prosome, macropain) subunit, alpha type, 2
PIAS1	26%	21%	E3-SUMO Ligase	protein inhibitor of activated STAT, 1 (DEAD/H box-binding protein 1)
MAK3	21%	16%	N-acetyltransferase	Mak3 homolog (S. cerevisiae)
PFAAP5	21%	21%	Immune?	phosphonoformate immuno-associated protein 5
MCP	**32%**	26%	Immune	membrane cofactor protein (CD46, trophoblast-lymphocyte cross-reactive antigen)
SMBP	26%	16%	Membrane protein	SM-11044 binding protein
MKLN1	21%	21%	Ischemic tolerance/Cell adhesion?	muskelin 1, intracellular mediator containing kelch motifs
ALDOA	26%	16%	Metabolism (glycolysis)	aldolase A, fructose-bisphosphate
IDI1	**32%**	26%	Metabolism	isopentenyl-diphosphate delta isomerase
CYB5-M	26%	16%	Metabolism	cytochrome b5 outer mitochondrial membrane precursor
GLO1	**37%**	21%	Metalloglutathione (GSH) transferase	glyoxalase I
EIF3S6	21%	16%	Translation	eukaryotic translation initiation factor 3, subunit 6 48kDa
EIF1AX	16%	21%	Translation	eukaryotic translation initiation factor 1A
PCBP2	16%	16%	Translation	poly(rC) binding protein 2 (hnRNPE2)
HNRPA2B1	32%	21%	Cell proliferation?	heterogeneous nuclear ribonucleoprotein A2/B1
CDK6	16%	16%	Cell-cycle	cyclin-dependent kinase 6
CCNE2	16%	16%	Cell-cycle	G1/S-specific cyclin E2
PUM2	26%	21%	Meiosis/RNA-binding	pumilio homolog 2 (Drosophila)
TRA2A	16%	26%	RNA-binding/?	transformer-2 alpha (putative MAPK activating protein PM24)
ATXN2	16%	21%	? (but has RNA motif)	ataxin 2
GTF2IP1	21%	26%	Pseudogene	general transcription factor II, i, pseudogene 1
H41	**53%**	26%	?	hypothetical protein
C19orf13	26%	**37%**	?	family with sequence similarity 61, member A (FAM61A)
CNIH	26%	26%	?	cornichon homolog (TGAM77)
LOC400986	26%	26%	?	protein immuno-reactive with anti-PTH polyclonal antibodies (HEM1)
ANKRD17	21%	21%	?	ankyrin repeat domain 17 (breast cancer antigen NY-BR-16)
RHOBTB3	16%	16%	? (GTPase)	Rho-related BTB domain containing 3
? - Unknown or unidentified gene product function				

90 genes were identified to be both coexpressed with p68 and p72, and are arranged by function. For clarity all coexpressed gene products with a 30% or greater coexpression frequency correlation for either p68 or p72 are in **bold**.

**Figure 2:**
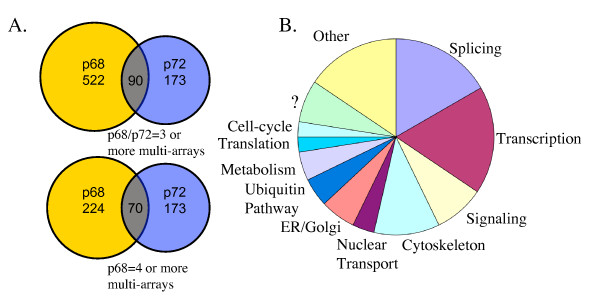
**Analysis of overlap of p68 and p72 coexpressed genes**. (A) Venn diagram of overlap of frequency = 3 or more, genes from p68 and p72 analysis, and when p68 frequency is increased to 4 or more. (B) Ontology pie-chart of p68/p72 overlapping frequency = 3 or more, gene products.

Even when the stringency was further augmented by increasing the p68 frequency cut-off to 4 or more multi-arrays (21% and above overlap within p68 multi-arrays), this lost almost 300 p68 hits, but only reduced the number of overlapping genes with p72 from 90 to 70 (Figure 2A). The highest frequency of overlap of p68 and p72 occurred in splicing, consistent with previous reports of their role in this process. Further validation of this technique was observed by the reciprocal gene hits of p68 and p72 (i.e. p72 was a positive for p68 and vice-versa), again consistent with their interaction within the same pathways.

The next most abundant function of p68 and p72 appeared to be in transcription (Figure 2B), once more consistent with previous reports. This is especially interesting given that p68 and p72 were previously shown to act as coactivators for the nuclear receptor estrogen receptor α (ERα) transcription factor, and we have identified X-box binding protein 1 (*XBP1*), associated with the ERα pathway. We have also identified 2 other nuclear receptor pathway proteins, the thyroid hormone receptor associated protein 2 (*THRAP2*) and the retinoic acid receptor-related orphan receptor α (*RORA*) transcription factor.

### RNA Helicase A(Dhx9) coexpresses and interacts with p68 and p72

A further interesting transcription-associated gene identified was RNA helicase A (*DHX9*), a member of a similar protein family to p68 and p72, all of which have been shown to interact with p300/CBP coactivators[6,11-13]. The frequency for both p68 and p72 were observed to be high for RNA helicase A (almost 50% of multi-arrays for p68, and over 30% for p72).

For this reason a similar coexpression analysis was separately performed for *DHX9*. Surprisingly, not only were p68 and p72 reciprocally coregulated with *DHX9*, but over 50% of the p68:p72 overlapped positives were also coexpressed with *DHX9 *(47 out of 90 – see Additional file 2). This was powerful evidence linking Dhx9, p68 and p72 to similar pathways.

**Figure 3:**
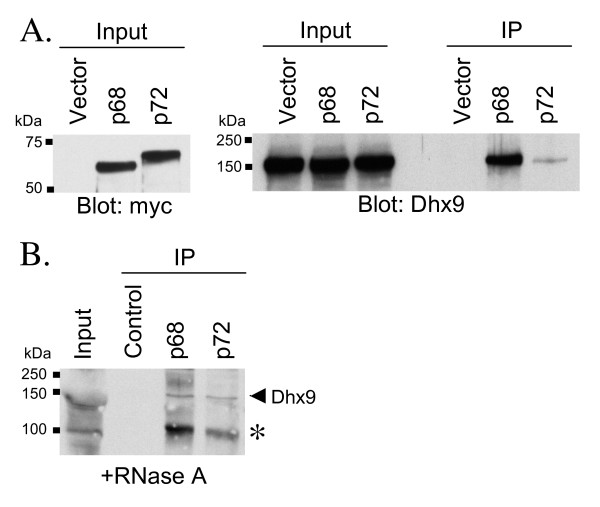
**p68 and p72 interact directly with predicted pathway partner Dhx9**. (A) Left panel shows myc immunoblot of inputs from transiently transfected myc-p68 or -p72, or vector alone. Right panel shows RNA helicase A (Dhx9) immunoblot of inputs and myc immunoprecipitations (IP). (B) Dhx9 immunoblot of endogenous IP of p68 and p72 from mouse liver lysate (RNase A pre-treated). * Indicates either a shorter Dhx9 isoform or a cross-reacting (but immuno-precipitating) protein.

As this overlap was so high it was possible that p68 and p72 were functioning in the same complex as Dhx9. This was tested experimentally in HEK293 cells. With immunoprecipitation of either transiently transfected p68 or p72 we observed a clear interaction with endogenous Dhx9 (figure 3A). Further imunoprecipitations of endogenous p68 and p72 from lysate of mouse liver confirmed the interaction with Dhx9 (figure 3B). This was performed after incubation with RNaseA, indicating a protein:protein interaction (as p68/p72/Dhx9 can all bind RNA). In the liver extract p68 and p72 also strongly immunoprecipitated a protein of 100 kDa, recognised by the Dhx9 antibody (figure 3B). It currently remains unclear if this is a different isoform of Dhx9 or a cross-reacting protein.

Altogether, these data both supported the hypothesis of p68/p72/Dhx9 existing within the same complex, and further acted as strong evidence of the predictive capabilities of the Oncomine™ analysis technique described here.

### Other coexpressed genes of p68 and p72

Interestingly, there were 4 overlapped hits in the ubiquitin pathway (and one proteasome) which may be related to the observation that p68 is highly ubiquitinated in colon cancers[14]. p68 was also recently shown to be SUMO modified, specifically SUMO-2 by PIAS1 ligase[15]. Here we shown that *PIAS1 *is coexpressed with p68/p72, and *SUMO-2 *is coexpressed with p68.

p68/p72 have also recently been shown to interact in a complex with ILF3, hnRNPU, and hnRNPH1 for microRNA processing[16]. Here, these gene products are also shown to be highly coexpressed with p68 and p72, supporting their role in the same complex/pathway (furthermore *DDX3X *is identified here with p68 and is also part of this microRNA processing complex).

In a separate study a group of proteins were identified in an mRNP complex with p68 and are here shown to be coexpressed with p68/p72 (*SFRS5*, *NFAR/ILF3*, *HNRNPA2/B1*, *HNRPU*, *PNN*, *TRA2A*, *DDX3X*) [17].

A new role for p68 and p72, suggested by our meta-analysis, might be in nuclear transport, given that a member of nuclear pore complex (Nup133) as well as nuclear import (transportin1) and export (exportin1) genes were identified as coexpressed genes.

Furthermore, coexpressed genes presented here are not limited to nuclear processes given that several cytoskeletal proteins are identified in the screening, implicating p68 and p72 in these processes (although probably indirectly as p68 and p72 are predominantly nuclear, perhaps acting via transcription or splicing). This is also true for endoplasmic reticulum (ER) or golgi proteins. Indeed, the *RAB6A *trafficking protein had the highest frequency overlap for p68 (almost 70% overlap), while being one of the lowest for p72 (16% overlap), possibly indicative of a functional difference between both. The family member *RAB14 *was also identified for both.

A further significant group of genes identified were involved in signal transduction, and may provide a start into analysis of regulation of p68 and p72 (although a meta-analysis like this can identify frequency of coexpression, it is impossible to say which protein may be regulating another, or indeed if both are targets of another protein).

Altogether the results of the overlapping coexpressed genes not only reiterate previous studies with either p68/p72 but predict new potential pathways in which p68/p72 may act.

### Selected non-overlapping coexpressed genes of p68 and p72

While p68 and p72 may be highly similar and involved in the same pathways, it remains likely that they are also involved in subtly different pathways. For this reason a similar ontology analysis was performed on genes that do not overlap between p68 and p72. However, given the extensive nature of the gene hits we selected all genes with frequency overlap above 30%, as well as some genes of interest from lower frequencies (Table 2).

**Table 2:** p68 and p72 frequency analysis of non-overlapping hits (all over 30% and selected below 30%).

p68 selected genes with no p72 overlap			
Gene	% Overlap	Function	Gene Name
FXR1	**42%**	RNA-binding/Unknown	fragile X mental retardation, autosomal homolog 1
HNRPK	**37%**	Transcription/Translation/Signaling	heterogeneous nuclear ribonucleoprotein K
NAP1L1	**32%**	Transcription	nucleosome assembly protein 1-like 1
JMJD1C	**32%**	Transcription	jumonji domain containing protein 1C (Thyroid receptor interacting protein 8)
SFRS11	**32%**	Splicing	splicing factor, arginine/serine-rich 11
MAPRE1	**37%**	Cytoskeleton	microtubule-associated protein, RP/EB family, member 1
ACTG2	**32%**	Cytoskeleton	actin, gamma 2, smooth muscle, enteric
PTPN11	**32%**	Signal Transduction	protein tyrosine phosphatase, non-receptor type 11 (Noonan syndrome 1)
JAK1	**32%**	Signal Transduction	janus kinase 1 (a protein tyrosine kinase)
ARF3	**32%**	Vesicular Trafficking	ADP-ribosylation factor 3
ANXA7	**32%**	ER-calcium mobilization	annexin A7 (Annexin VII) (Synexin)
COX7A2L	**32%**	Metabolism	cytochrome c oxidase subunit VIIa polypeptide 2 like
C6orf55	**32%**	Anti-metastatic protein	protein C6orf55 (Dopamine responsive protein DRG-1) (My012 protein)
LAPTM4A	**32%**	compartmentalization of amphipathic solutes	lysosomal-associated protein transmembrane 4 alpha
ZNF9	**32%**	?	zinc finger protein 9 (a cellular retroviral nucleic acid binding protein)
TDE1	**32%**	?	tumor differentially expressed 1
SYPL	**32%**	?	synaptophysin-like 1
NUCKS	**32%**	?	nuclear, casein kinase and cyclin-dependant kinase substrate
ELK3	26%	Transcription	ELK3, ETS-domain protein (SRF accessory protein 2)
THRAP1	21%	Transcription	thyroid hormone receptor associated protein 1
RBBP4	21%	Transcription	retinoblastoma binding protein 4 (chromatin assembly factor/CAF-1 p48 subunit)
ERBP	21%	Transcription	estrogen receptor binding protein
RARA	16%	Transcription	retinoic acid receptor, alpha
HDAC2	16%	Transcription	histone deacetylase 2
SNRPB	26%	Splicing	small nuclear ribonucleoprotein polypeptides B and B1
TAPBP	26%	ER chaperone/Protein folding	TAP binding protein (tapasin)
CALR	21%	ER chaperone/Protein folding	calreticulin
CANX	16%	ER Chaperone/Protein folding	calnexin
RAB1A	26%	ER-golgi Transport	RAB1A, member RAS oncogene family
RAB11B	21%	Membrane recycling	RAB11B, member RAS oncogene family
UCHL1	21%	Ubiquitin	ubiquitin carboxyl-terminal esterase L1 (ubiquitin thiolesterase)
PSMA2	21%	Proteolysis	proteasome (prosome, macropain) subunit, alpha type, 2
PRKWNK1	21%	Proteolysis	proteasome (prosome, macropain) 26S subunit, non-ATPase, 1
SUMO2	16%	SUMO pathway	small ubiquitin-like modifier, 2
CDC42	21%	Cell-cycle	cell division cycle 42 (GTP binding protein, 25kDa)
CDC40	21%	Cell-cycle	cell division cycle 40 homolog (yeast) [pre-mRNA splicing factor 17]
CDC10	26%	Cytokinesis?	septin-7 (CDC10 protein homolog)
LITAF	26%	p53-induced Apoptosis	lipopolysaccharide-induced TNF factor (p53-induced protein 7)
EIF3S10	26%	Translation	eukaryotic translation initiation factor 3, subunit 10 theta, 150/170 kDa

p72 selected genes with no p68 overlap			
**Gene**	**% Overlap**	**Function**	**Gene Name**
TTC3	**32%**	?	tetratricopeptide repeat domain 3
HMGN4	26%	Transcription	high mobility group nucleosomal binding domain 4
CTBP1	21%	Transcription/corepressor	C-terminal binding protein 1
MTA1	21%	Transcription/ER-alpha repressor	metastasis associated 1
HDAC7A	16%	Transcription	histone deacetylase 7A
NONO	16%	Splicing/Transcription	non-POU domain containing, octamer-binding (p54nrb)
SFRS3	16%	Splicing	splicing factor, arginine/serine-rich 3
MAP2K3	26%	Signal Transduction	mitogen-activated protein kinase kinase 3
ERBB3	16%	Signal Transduction	receptor protein-tyrosine kinase erbB-3
CSK	16%	Signal Transduction	c-src tyrosine kinase
CALM2	16%	Signal Transduction	calmodulin 2 (phosphorylase kinase, delta)
RPS6	21%	Ribosome	40S ribosomal protein S6
RPS15A	21%	Ribosome	40S ribosomal protein S15a
MRPS6	26%	Mitochondrial Ribosome Protein	mitochondrial ribosomal protein S6
PABPC1	26%	Translation	poly(A) binding protein, cytoplasmic 1
EIF5	21%	Translation	eukaryotic translation initiation factor 5
EDD	26%	ubiquitin E3 Ligase	ubiquitin--protein ligase EDD
ARPC3	26%	Cytoskeleton	actin related protein 2/3 complex, subunit 3,21 kDa
WSB1	21%	?	WD repeat and SOCS box-containing 1
GARNL1	21%	?	GTPase activating RANGAP domain-like 1
?-Genes with unknown function. Genes with > 30% frequency overlap are in **bold**.			

All coexpressed but non-overlapping gene products for p68 and p72 over 30% frequency are shown (and are in **bold**). Selected coexpressed gene products below 30% are shown and were chosen based on interest and common ontology groups.

For p68 the genes above 30% generally fell into the same categories as previously, while there was only 1 gene identified for p72, with no obvious molecular function. Of-course the selected genes below 30% were chosen based on interest and common ontological groupings, and may not be representative. However, we note that for p68 more RAB family members are identified (*RAB1A*, *RAB11B*) as well as more ER proteins, particularly protein folding chaperones (Tapasin, Calnexin, Calreticulin).

With regard to transcription, p68 coexpressed with *ELK3 *and *HDAC2 *transcriptional repressors, while p72 coexpressed with *CTBP1 *and *HDAC7 *repressors. This might be relevant given that p68 and p72 have been shown to act as transcriptional repressors, hypothesised to have different mechanisms of action as they act in a promoter-specific manner[7]. However it has been shown that CTBP1 repressive function is antagonized by pinin[18], and here, both p68 and p72 also coexpress with pinin (*PNN*)[17]. p68 has also been shown to be involved in p53 coactivation[4], and here we identify a coexpressed p53 coactivator hnRNPK[19] for p68/p72 and the p53-induced protein 7 (*LITAF*), for p68. For other transcription roles for p68 there were more nuclear receptor pathway proteins including thyroid receptor interacting protein 8 (*JMJD1C*),*THRAP1 *(*THRAP2 *was identified above for both p68 and p72), estrogen receptor binding protein (*ERBP*), and the retinoic acid receptor alpha (*RARA*) transcription factor. p72 coexpressed with the ER-alpha repressor *MTA1*. We have also observed that p68 coexpressed gene *ZNF9 *is in the same pathway as p68/p72 coexpressed *MBNL1*, implicated in myotonic dystrophy[20].

For p72 we note that NonO (p54nrb) has been shown to interact with SFPQ/PSF[21] (*SFPQ *identified as coexpressed for both p68 and p72). Furthermore EDD (a ubiquitin E3 ligase), also identified here with p72, has been shown in a complex with SFPQ[22]. Remarkably p68 has also very recently been shown to interact in a complex with NonO and SFPQ/PSF[23], again confirming the validity of the technique described here.

## Discussion

The technique described here has proven useful in increasing the stringency of Oncomine™ meta-analysis, and will prove to be widely applicable. Generally individual gene levels cannot be compared from one study to another, but the strength of our analysis is an inter-study comparison (meta-analysis) after an intra-study Oncomine™ analysis (coexpression gene search).

While we still retain the strongest 400 coexpressed genes from each multi-array, it becomes de-sorted when analyzing for frequency over different studies. An example is *EDEM1 *(involved in protein folding in the ER), which is consistently one of the strongest correlated genes with p72, while having only a 32% frequency overlap. The same is true for p68 and Sp3 transcription factor with a frequency overlap of 37%, and very highly coexpressed in these individual studies. Conversely, the technique described here is useful for comparison of coexpressed genes which may not always have a high coexpression coefficient, giving another advantage over analysis of single studies.

An interesting exception is *RAB6A *with p68 which has both the highest frequency overlap with p68 (68%) and is almost always within the first 100 genes coexpressed with p68 in individual multi-array studies. A further exception is RNA helicase A (*DHX9*) which again has a high frequency of overlap with p68 (47%) and usually is within the first 50 coexpressed genes with p68. We have also shown here for the first time an interaction by immunoprecipitation of p68 (and also p72), with Dhx9.

Furthermore, the technique described here is most useful in clustering specific genes involved in pathways when meta-analysis hits from known interacting proteins can be overlapped. We observed with our example of p68 and p72 that the overlapping hits mainly clustered into the classes of ontology in which p68/p72 had already been reported, namely splicing and transcription, further acting as validation for this type of analysis.

While some new proposed pathways for p68/p72 cannot be through direct action (e.g. cytoskeletal remodelling or ER-protein folding) it remains possible that p68/p72 are involved in these pathways indirectly via splicing/transcription/controlling nuclear shuttling. We were encouraged by the fact that p68 and/or p72 coexpressed with previously published interacting proteins such as one-another, ILF3, hnRNPH1, hnRNPU, hnRNPA2/B1, SFRS5, Ddx3X, PIAS1, SUMO2, pinin, NonO and SFPQ and were further encouraged by observation of coexpression with members of pathways in which they were previously shown to act, such as estrogen receptor pathway (*XBP1*, *MTA1*, *ERBP*, *DDX5*, *DDX17*), ubiquitin pathway (*USPX9*, *UBE2J1*, *UBE3A*, *BIRC6*, *UCHL1*, *EDD*), translation (*EIF3S6*, *EIF1A*, *EIF3S10*, *PABPC1*, *EIF5*), and transcriptional repression (*HDAC2*, *HDAC7A*, *PNN*, *ELK3*, *CTBP1*, *MTA1*).

There also seems to be a more general role for p68 and p72 in nuclear receptor transcription pathways than first assumed (ERα pathway as above), for example *JMJD1C*, *THRAP1*, *THRAP2*, *RARA*, *RORA*, all coexpress with p68 and/or p72.

While it is clear that we have obtained a highly stringent list of potential pathway partners of p68 and p72, with regard to separable functions (i.e. non-overlapping genes of p68 and p72) we cannot say with confidence as genes generally clustered into the same pathways as for the overlapping list. This may be due to a high false-negative rate of this technique as we have used several levels of stringency, and will most likely exclude many true pathway partners of p68 and p72. However, this cost is offset by high quality results using our rigorous analysis.

## Conclusion

It is apparent that we have increased the scope of the Oncomine™ database, by utilising frequency of coexpression (meta-analysis) over different multi-array studies to predict pathway partners of searched proteins. With regard to the p68 and p72 RNA helicases we have identified a non-exhaustive list of gene products that are likely to be present in various pathways in which p68 and/or p72 act, both corroborating previous studies and making novel predictions. For one of these, RNA helicase A(Dhx9), we have shown there is a direct interaction with p68 and p72. Future experimental studies using this list as a reference point will reveal the validity of this technique.

## Methods

### Oncomine analysis

The following procedure was undertaken for meta-analysis (figure 1B):

(1) Oncomine™ expression correlations were searched for p68 (*DDX5*) or p72(*DDX17*). (2) 19 different mult-arrays were chosen and the first 400 correlated genes within each multi-array were compared using Microsoft Excel, (separately for p68 and p72). Importantly, repetitive genes were then removed within each study, leaving only 1 representative per multi-array study. When a coregulated gene appeared in more than 3 multi-array experiments it was accepted as significant (3 = 16% frequency of the 19). These genes were taken as more significant than analysis of a single Oncomine™ output. Furthermore, given that the user cannot choose which multi-array will be given by Oncomine™ there was no attempt to specifiy different tissue types or cancer types. This had the advantage of giving a more generalised result of which pathways the proteins may be involved in, which was preferred for an initial study such as that performed here. (3) These sorted lists of coregulated genes given for p68 and p72 were compared for overlapping genes which added another level of stringency, and greatly increased the significance of the results. The genes listed were then investigated for ontology, and full gene/gene-product names, using a combination of Pubmed searches[24], Fatigo[25], and Genecards[26].

### Cell culture, transfection, immunoprecipitation and western blot

HEK293 cells were transfected with either pSG5-myc, pSG5-myc-p68, pSG5-myc-p72 (plasmids were a gift from Frances Fuller-Pace, Dundee, UK), using FuGENE 6 (Roche). 48 h post-transfection cells were harvested on ice in buffer B (150 mM KCl, 0.1% NP-40, 20 mM Tris-HCl pH8.0, 5 mM MgCl_2_, 10% glycerol, 5 mM NaF, 1× Roche complete protease inhibitor cocktail). 600 μg of total cell extract was incubated with 5 μg 9E10 anti-myc monoclonal antibody, and protein G sepharose (GE Healthcare), rotating at 4°C for 2 h. Pellet was washed 3× in buffer B, boiled in protein loading buffer that was then run on an SDS-PAGE gel, transferred to pvdf and immunoblotted overnight at 4°C for Dhx9 (Bethyl Laboratories) or myc.

For endogenous co-immunoprecipitation liver was extracted from a 3 mth old male mouse and homogenised in buffer B (Brinkmann polytron). Lysis was allowed to happen, rotating at 4°C for 30 min. Sample was then centrifuged to remove debris and further incubated with RNaseA, rotating at 4°C for 30 additional minutes, while preclearing lysate with protein G sepharose. 2 mg of this lysate was used with 3 μg of either p68 or p72 antibodies (Bethyl Laboratories) per immunoprecipitation, which were performed as above.

## Authors' contributions

BJW conceived and designed the study, analyzed the data, performed the co-immunoprecipitation experiments, and wrote the manuscript. VG critically reviewed the manuscript and approved the final version.

## Supplementary Material

Additional file 1 p68(*DDX5*) and p72(*DDX17*) coexpressed genes. Table of all coexpressed genes of p68 and p72 (individual analyses) with frequency cutoff of 3 multi-array studies. Click here for file

Additional file 2 *DHX9 *coexpressed genes and overlaps with p68(*DDX5*) and p72(*DDX17*). Table of *DHX9 *Oncomine meta-analysis for coexpressed genes. Frequency cutoff of 3 multi-array studies. Overlap with p68 and p72 individual coexpression gene lists is shown, as is the overlap with the p68:p72 common gene list. Click here for file

## References

[B1] Oncomine http://www.oncomine.org

[B2] Rhodes DR Kalyana-Sundaram S Mahavisno V Barrette TR Ghosh D Chinnaiyan AM Mining for regulatory programs in the cancer transcriptome Nat Genet 2005 37 6 579 583 10.1038/ng1578 15920519

[B3] Ogilvie VC Wilson BJ Nicol SM Morrice NA Saunders LR Barber GN Fuller-Pace FV The highly related DEAD box RNA helicases p68 and p72 exist as heterodimers in cells Nucleic Acids Res 2003 31 5 1470 1480 12595555 10.1093/nar/gkg236 PMC149829

[B4] Bates GJ Nicol SM Wilson BJ Jacobs AM Bourdon JC Wardrop J Gregory DJ Lane DP Perkins ND Fuller-Pace FV The DEAD box protein p68: a novel transcriptional coactivator of the p53 tumour suppressor Embo J 2005 24 3 543 553 15660129 10.1038/sj.emboj.7600550 PMC548656

[B5] Metivier R Penot G Hubner MR Reid G Brand H Kos M Gannon F Estrogen receptor-alpha directs ordered, cyclical, and combinatorial recruitment of cofactors on a natural target promoter Cell 2003 115 6 751 763 10.1016/S0092-8674(03)00934-6 14675539

[B6] Watanabe M Yanagisawa J Kitagawa H Takeyama K Ogawa S Arao Y Suzawa M Kobayashi Y Yano T Yoshikawa H A subfamily of RNA-binding DEAD-box proteins acts as an estrogen receptor alpha coactivator through the N-terminal activation domain (AF-1) with an RNA coactivator, SRA Embo J 2001 20 6 1341 1352 11250900 10.1093/emboj/20.6.1341 PMC145523

[B7] Wilson BJ Bates GJ Nicol SM Gregory DJ Perkins ND Fuller-Pace FV The p68 and p72 DEAD box RNA helicases interact with HDAC1 and repress transcription in a promoter-specific manner BMC Mol Biol 2004 5 11 15298701 10.1186/1471-2199-5-11 PMC514542

[B8] Guil S Gattoni R Carrascal M Abian J Stevenin J Bach-Elias M Roles of hnRNP A1, SR proteins, and p68 helicase in c-H-ras alternative splicing regulation Mol Cell Biol 2003 23 8 2927 2941 12665590 10.1128/MCB.23.8.2927-2941.2003 PMC152554

[B9] Honig A Auboeuf D Parker MM O'Malley BW Berget SM Regulation of alternative splicing by the ATP-dependent DEAD-box RNA helicase p72 Mol Cell Biol 2002 22 16 5698 5707 12138182 10.1128/MCB.22.16.5698-5707.2002 PMC133985

[B10] Li J Hawkins IC Harvey CD Jennings JL Link AJ Patton JG Regulation of alternative splicing by SRrp86 and its interacting proteins Mol Cell Biol 2003 23 21 7437 7447 14559993 10.1128/MCB.23.21.7437-7447.2003 PMC207616

[B11] Rossow KL Janknecht R Synergism between p68 RNA helicase and the transcriptional coactivators CBP and p300 Oncogene 2003 22 1 151 156 10.1038/sj.onc.1206067 12527917

[B12] Warner DR Bhattacherjee V Yin X Singh S Mukhopadhyay P Pisano MM Greene RM Functional interaction between Smad, CREB binding protein, and p68 RNA helicase Biochem Biophys Res Commun 2004 324 1 70 76 10.1016/j.bbrc.2004.09.017 15464984

[B13] Nakajima T Uchida C Anderson SF Lee CG Hurwitz J Parvin JD Montminy M RNA helicase A mediates association of CBP with RNA polymerase II Cell 1997 90 6 1107 1112 10.1016/S0092-8674(00)80376-1 9323138

[B14] Causevic M Hislop RG Kernohan NM Carey FA Kay RA Steele RJ Fuller-Pace FV Overexpression and poly-ubiquitylation of the DEAD-box RNA helicase p68 in colorectal tumours Oncogene 2001 20 53 7734 7743 10.1038/sj.onc.1204976 11753651

[B15] Jacobs AM Nicol SM Hislop RG Jaffray EG Hay RT Fuller-Pace FV SUMO modification of the DEAD box protein p68 modulates its transcriptional activity and promotes its interaction with HDAC1 Oncogene 2007 26 40 5866 5876 10.1038/sj.onc.1210387 17369852

[B16] Gregory RI Yan KP Amuthan G Chendrimada T Doratotaj B Cooch N Shiekhattar R The Microprocessor complex mediates the genesis of microRNAs Nature 2004 432 7014 235 240 10.1038/nature03120 15531877

[B17] Merz C Urlaub H Will CL Luhrmann R Protein composition of human mRNPs spliced in vitro and differential requirements for mRNP protein recruitment Rna 2007 13 1 116 128 17095540 10.1261/rna.336807 PMC1705747

[B18] Alpatov R Munguba GC Caton P Joo JH Shi Y Shi Y Hunt ME Sugrue SP Nuclear speckle-associated protein Pnn/DRS binds to the transcriptional corepressor CtBP and relieves CtBP-mediated repression of the E-cadherin gene Mol Cell Biol 2004 24 23 10223 10235 15542832 10.1128/MCB.24.23.10223-10235.2004 PMC529029

[B19] Moumen A Masterson P O'Connor MJ Jackson SP hnRNP K: an HDM2 target and transcriptional coactivator of p53 in response to DNA damage Cell 2005 123 6 1065 1078 10.1016/j.cell.2005.09.032 16360036

[B20] Kino Y Mori D Oma Y Takeshita Y Sasagawa N Ishiura S Muscleblind protein, MBNL1/EXP, binds specifically to CHHG repeats Hum Mol Genet 2004 13 5 495 507 10.1093/hmg/ddh056 14722159

[B21] Emili A Shales M McCracken S Xie W Tucker PW Kobayashi R Blencowe BJ Ingles CJ Splicing and transcription-associated proteins PSF and p54nrb/nonO bind to the RNA polymerase II CTD Rna 2002 8 9 1102 1111 12358429 10.1017/S1355838202025037 PMC1370324

[B22] Zhang C Dowd DR Staal A Gu C Lian JB van Wijnen AJ Stein GS MacDonald PN Nuclear coactivator-62 kDa/Ski-interacting protein is a nuclear matrix-associated coactivator that may couple vitamin D receptor-mediated transcription and RNA splicing J Biol Chem 2003 278 37 35325 35336 10.1074/jbc.M305191200 12840015

[B23] Liang S Lutz CS p54nrb is a component of the snRNP-free U1A (SF-A) complex that promotes pre-mRNA cleavage during polyadenylation Rna 2006 12 1 111 121 16373496 10.1261/rna.2213506 PMC1370891

[B24] Pubmed http://www.ncbi.nlm.nih.gov/sites/entrez?db=pubmed

[B25] Fatigo http://fatigo.bioinfo.cipf.es/

[B26] Genecards http://www.genecards.org

